# Ethico-Political Aspects of Conceptualizing Screening: The Case of Dementia

**DOI:** 10.1007/s10728-021-00431-3

**Published:** 2021-03-16

**Authors:** Martin Gunnarson, Alexandra Kapeller, Kristin Zeiler

**Affiliations:** 1 Department of Thematic Studies, Division of Technology and Social Change, Linköping University, Linköping, Sweden; 2 Centre for Studies in Practical Knowledge, School of Culture and Learning, Södertörn University, Huddinge, Sweden

**Keywords:** Screening, Dementia, Case-finding, Conceptualizations, Performativity

## Abstract

While the value of early detection of dementia is largely agreed upon, population-based screening as a means of early detection is controversial. This controversial status means that such screening is not recommended in most national dementia plans. Some current practices, however, resemble screening but are labelled “case-finding” or “detection of cognitive impairment”. Labelled as such, they may avoid the ethical scrutiny that population-based screening may be subject to. This article examines conceptualizations of screening and case-finding. It shows how the definitions and delimitations of the concepts (the *what* of screening) are drawn into the ethical, political, and practical dimensions that screening assessment criteria or principles are intended to clarify and control (the *how* of screening, how it is and how it should be performed). As a result, different conceptualizations of screening provide the opportunity to rethink what ethical assessments should take place: the conceptualizations have different ethico-political implications. The article argues that population-based systematic screening, population-based opportunistic screening, and case-finding should be clearly distinguished.

## Introduction

The hunt for dementia can’t be called a screening programme because it would not meet the standards of the UK National Screening Committee. But call it case finding and suddenly there’s no need for evidence that it protects the public against false positives and negatives or society from the injustice of more resources directed towards the least unwell [21].

What McCartney calls the “hunt for dementia” is motivated by an understanding of dementia as one of the major challenges facing healthcare systems. The incidence and prevalence of the condition are expected to increase dramatically in coming years, as are also the societal costs and human suffering [2, 4].

Population-based dementia screening is debated in scientific and political contexts worldwide [see e.g. 2,7], as one possible route to the generally accepted goal of diagnosing dementia earlier [2, 4, 17, 36]. This debate is characterized by a lively *conceptual* discussion and experimentation,^1^ which, we argue in this article, has ethico-political implications. In the United Kingdom, the National Screening Committee has said “No” to population-based screening for dementia, but a “case-finding” scheme for dementia has been launched [8, 9]. As McCartney states in the quotation above, labelling this practice “case-finding” might entail less strict assessments of its effects on patients and society. Similarly, population-based screening has not been recommended in the USA, but the publicly funded Annual Wellness Visit includes a “detection of cognitive impairment”.

The first aim of this article is to analyze different conceptualizations of screening, and related terms, with a focus on how distinctions and delimitations open up different possibilities for action. We examine the relationship between concepts such as “screening” and “case-finding” and the practices they denote, as a relationship between what we call the *what* and the *how* of screening. The *what* denotes the definitions and delimitations of the screening and case-finding concepts. The *how* denotes a wide range of aspects, from the criteria formulated for how screening and screening-like practices should be performed to the actual administration of a screening test. The second aim of the article is based on this analysis: to help reduce the lack of clarity from which screening and related concepts suffer by offering a set of recommendations.

## The “What” and the “How”

We understand concepts and practices to be intimately linked in a dynamic and performative relationship, rather than neutral descriptions of an already existing reality. Through their relationship to each other, concepts and practices help create and shape each other.

This understanding has been inspired by Ian Hacking, who has shown how concepts and classifications help to create “kinds of people” [14]. Rather than naming categories that already exist, scientific concepts, according to Hacking, “may bring into being a new kind of person, conceived of and experienced as a way to be a person” [14]. Among his examples are multiple personality disorder and autism, whose emergence as medical diagnoses, he argues, gave rise to two new ways of being a person. However, he points out, the relationship between concepts and people is not stable and universal: it is dynamic and contextual [13]. While concepts can bring ways of being a person into being, such that people identify with the concepts, people can in return transform the concepts through their actions. There is thus a “looping effect” between concepts and the ways of being a person that they help to create, through which they affect and shape each other [14].

Our focus here is less on the relationship between concepts and people, and more on the relationship between concepts and practices. However, in stating that both “our selves” and “our spheres of possibility” may be “made up by our naming and what that entails”, Hacking suggests not only that what we can *be,* but also what we can *do,* stand in a close relationship to conceptualizations [13]. As a result, neither conceptualizations nor practices are neutral: through their intimate relationship, they make some realities rather than others possible. They are ethico-politically charged, since they involve deliberations about what actions are right and what kind of society we should have.

The conceptualization and design of screening practices must take this dynamic relationship between concepts (the *what*) and practices (the *how*) into account. If it is left unexamined, the ethico-political aspects and consequences of particular concepts and practices, and their relation, may be overlooked. The present article, therefore, has the normative vision to contribute to an increased awareness of this dynamic relationship and, in doing so, help reduce the lack of clarity characterizing current screening concepts and practices. Thus, even though the interplay between concepts and practices is never stable nor neutral, this article departs from the view that it can and should be scrutinized and acted upon. Therefore, we end the text with a set of recommendations for how some screening concepts and practices should be linked in order that their ethico-political aspects and consequences can be carefully considered. The recommendations are based on our analysis of the case of screening for dementia and should not be seen as exhaustive, but they do have bearing on several other forms of screening.

The relationship between the *what* and the *how* of screening can be illustrated with an important example. In 1968, the World Health Organization published James Wilson and Gunnar Jungner’s report *Principles and Practice of Screening for Disease*. The report formulated ten principles for assessing screening programs and contained definitions of screening and related concepts. For their understanding of screening, Wilson and Jungner relied on a report from the US Commission on Chronic Illness, which defined screening as “the presumptive identification of unrecognized disease or defect by the application of tests, examinations, or other procedures which can be applied rapidly” [30, quoted in 35]. Screening, Wilson and Jungner stated, is concerned with “unrecognized symptomatic” and “pre-symptomatic disease” (ibid.). It may include a variety of procedures (physical examinations, questionnaires and even “diagnostic” tests), provided that these can be administered rapidly. “In general,” they concluded, “we have taken the definition [of screening] to imply a relatively simple (though not necessarily unsophisticated) method of case-finding” (ibid.).

Wilson and Jungner’s conceptualization of screening (the *what*) was inclusive. In their definition, even diagnostic tests and case-finding could be considered to be screening. They also discussed the *how* of screening at length, and they formulated ten principles, or criteria, that must be met in order for screening to be justifiably offered. These criteria specified not only that “there should be a suitable [screening] test or examination”, but also, for example, that “there should be an accepted treatment for patients with recognized disease”, and that “facilities for diagnosis and treatment should be available” [35]. Thus, Wilson and Jungner defined demanding conditions for *how* screening should be practised. This *how* has had a profound impact on *what* screening can be today, long after the report was published.

Since 1968, Wilson and Jungner’s criteria have been central to screening program assessment. They are sometimes referred to as the “gold standard”, even if new criteria have been added [1]. Present-day criteria typically not only target the administration of the screening test, but also specify conditions that must be in place before screening activities can start (e.g. “there should be evidence that the complete screening programme (test, diagnostic procedures, treatment/intervention) is clinically, socially and ethically acceptable to health professionals and the public”), and specify what should happen after the test has been administered (e.g. “there should be an agreed policy on the further diagnostic investigation of individuals with a positive test result and on the choices available to those individuals”) [see 27].

In present-day definitions, screening is typically conceptualized as an activity that is offered to everyone in a predefined population, as in the UK National Screening Committee’s definition, where screening is:

a public health service in which members of a defined population, who do not necessarily perceive they are at risk of, or are already affected by, a disease or its complications, are asked a question or offered a test to identify those individuals who are more likely to be helped than harmed by further tests or treatment to reduce the risk of disease or its complications [25].

This implies a focus on population-based screening, which is also what national screening committees typically assess. In this way, Wilson and Jungners’ *how* of screening (with any criteria added later) has come to be associated with a particular *what*, namely population-based screening. Population-based screening, typically, is required to follow the gold-standard statements and newer amendments about *how* screening should be performed. This situation has spurred a conceptual discussion and experimentation, in which *the concept of screening is redefined, different types of screening are distinguished* (such as when “population-based screening” is distinguished from “opportunistic screening”), or *a distance from the concept of screening* is sought (such as when it is emphasized that “active” and “passive” case-finding should be understood as different from screening).^2^ Further, while a frequently used set of criteria has been defined for the assessment of population-based screening, no similar set exists for other types of screening or the various forms of case-finding. Below, we discuss the implications of these conceptualizations using the example of dementia screening and the theoretical analytic tools of the *what* and the *how*.

## The Work of Distinctions and Demarcations

Screening for dementia is but a first step that can lead to a dementia diagnosis, and it is performed before an early diagnosis of dementia. However, calls for early detection of dementia may resonate well with screening: the value attributed to earliness is present in both cases.^3^ At the same time, national screening boards typically do not recommend screening for dementia [see, for example, 15,24].^4^ Even so, because of the widely-shared ambition to achieve early detection, various forms of intervention have been proposed, and a wide variety of conceptualizations of these interventions has been put forward. To complicate things further, the same test to assess cognitive impairment can be used both in case-finding and in screening (such as the Mini Mental State Examination and the Abbreviated Mental Test Scale) [5, 26].

### Population-Based Screening Versus Screening Tests

While there is a consensus about the value of diagnosing dementia early, arguments are often raised against screening for dementia. Boustani et al., for instance, state that while some screening tests have “reasonable accuracy for detecting mild to moderate dementia”, uncertainties remain about the potential harms and benefits of population-based screening for dementia, and, most importantly, about the effectiveness of treatments for those whose dementia would be detected this way [7]. The lack of evidence concerning the potential harms and benefits of a population-based screening program for dementia is also central to why national bodies in the UK and the United States have advised against this form of screening [28].

Ashford et al. are an exception in these discussions. They argue for the introduction of a population-based screening program for dementia [2, 3]. This makes these authors particularly interesting for our analysis: What makes this argument possible in the face of the wide criticism of screening for dementia?

Central to the argument Ashford et al. put forward is a sharp distinction between the initial screening test and later diagnostic procedures—a distinction that Wilson and Jungner did not make. Based on this distinction, Ashford et al. state that “it is legitimate to insist that screening tests be properly evaluated”, and that screening should not be “asked to bear the responsibility for negative consequences associated with a lack of available clinical expertise, supervision, and counselling once dementia is identified” [2]. They propose a “new operating definition of dementia screening” (ibid.), in which the term “screening” refers only to the initial test. Negative implications of later diagnostic procedures, such as false-positive diagnoses, overdiagnosis or any negative effects of clinical treatment decisions, are not reason enough to advise against screening. In their words: the “absence of empirical data on the specific impact of screening on patient outcomes is not sufficient to justify a decision to recommend against it” (ibid.).

In narrowing down the definition of *what* screening is, conceptually delimiting it to the application of the test, Ashford et al. open up the possibility to distance themselves from and thereby re-evaluate the generally accepted screening criteria that state *how* screening should be performed. This allows them to argue that not only should the criteria be limited to the initial testing, but they should also be adapted to the particular circumstances of the disease in question, in their case dementia. Through these steps, they argue that population-based screening for dementia is justified.

Whereas Ashford et al. are among the few scholars who explicitly argue for this “new” understanding of screening, their suggestion ties into a conceptual lack of clarity. We note a tendency here: the line between the terms “screening” and “screening test” (or “screening tool” or “screening instrument”) is frequently blurred. While screening assessments in national settings include much more than the assessment of various screening tests, several examples can be found in the literature in which screening test assessment is conflated with screening assessment. This is most evident in publications that discuss and compare the efficacy of various screening tests without taking into account their long-term harms and benefits, later subsequent diagnostics, possible treatments, and so on. Even so, these assessments are used to indicate the viability of screening in general [3, 16, 22, 36]. While it is clearly the case that the various screening tests must be assessed with regard to their accuracy, specificity and sensitivity, this use of the concepts (in which screening test assessment is a form of screening assessment) may cause the phenomenon of screening to become primarily associated with the application of particular instruments. If this occurs, a re-evaluation of screening assessment criteria may be justified.

### Case-Finding, Cognitive Impairment Assessment and Opportunistic Screening

While Wilson and Jungner equated the concept of screening with case-finding, a distinction between the two concepts has arisen in recent years. The UK “case-finding” scheme for dementia exemplifies this. From 2013, all persons above the age of 75 who are admitted to a hospital in England, unplanned and for more than 72 h, have been asked a “case-finding question” about their memory, after which a test intended to help identify cognitive impairment may follow [34]. Even though this practice has several characteristics of screening (as defined by the UK National Screening Committee), the most notable of which is that it targets “members of a defined population, who do not necessarily perceive they are at risk of, or are already affected by, a disease or its complications”, it is termed “case-finding” in the official terminology [34].

Another example of a “screening-like” practice for which the term “screening” is not used is the “detection of cognitive impairment” that takes place as a part of the Medicare Annual Wellness Visit (AWV) in the US. The AWV is offered to all US citizens above the age of 65. During the visit, cognitive function is assessed in a procedure in which the physician observes and interviews the patient together with a knowledgeable informant (family member, friend, caregiver, other) about potential cognitive concerns. “If appropriate”, the regulations issued by the Centers for Medicare & Medicaid Services (CMS) state, “use a brief validated structured cognitive assessment tool” [11]. However, the regulations do not provide any guidance about how to determine when the use of a cognitive assessment tool is appropriate [19].^5^ Despite this uncertainty, this practice, just as the UK “case-finding” scheme, has several of the features of screening: it targets a predefined, pre-symptomatic population and involves control questions and/or the use of a brief test instrument. Even so, in the official terminology, the cognitive assessment that takes place during the AWV is referred to as “detection of cognitive impairment” or “cognitive impairment assessment” [10, 12].

So why is the term “screening” not used in these cases? A simple answer is that screening for dementia is not supported by the national health authorities in the two countries [18, 24]. It would therefore probably be next to impossible to introduce such a program, especially a population-based screening program, on a national scale. In both countries, however, the pressure to improve (early) detection and diagnosis of dementia has been high. This has caused politicians to act and launch programs such as those described above [19, 34]. However, in order to do so without contradicting the recommendation of the national health authorities, these programs cannot be called screening. They must be given other names.

What takes place here is a conceptual *distancing from the concept of screening*. Rather than arguing for a *redefinition* of *what* screening is, as Ashford et al. do, the message is that we are concerned with a completely different *what*, namely that of “case-finding” or “detection of cognitive impairment”. A deeper understanding of this conceptual approach can be gained by viewing it through the relationship between the *what* and the *how*. Due to the intimate bond between the *what* and *how* of screening, a distance from the concept of screening may enable a distancing from the demanding assessment criteria so closely associated with it. In other words, giving the practice another name may make it possible to introduce screening-like practices without the need to abide by the established criteria.

A generally accepted definition of case-finding has not been established, nor have any generally accepted assessment criteria for the process been laid down [28]. The *what* and *how* of case-finding are thus more “open” than those of screening. As a result, a wide variety of practices can be labelled “case-finding”, and it is possible to distinguish several forms of it. As Ranson et al. [28] express it: “there is much ambiguity around what it [case-finding] means, particularly with respect to how it differs from screening”. They suggest that case-finding should be understood as:

an offer of a brief, opportunistic investigation to identify possible signs or symptoms of dementia, initiated by a clinician during consultation with a patient at high risk of dementia on the basis of clinical judgment that an initial dementia enquiry is appropriate and is likely to benefit the patient [28].

Ranson et al. suggest that two distinguishing features be used to define case-finding. First, case-finding does not, as screening does, target a predefined population, but is applied on a case-by-case basis. Second, the determination of whether case-finding should be applied or not is the result of an individual clinician’s clinical judgment about whether the procedure “is appropriate and is likely to benefit the patient”, not the result of identifying a particular population to whom the test is offered [28].

With Ranson et al., the possibility of introducing screening-like practices by naming them “case-finding” vanishes. In their conceptualization, the *what* of case-finding is filled with a particular content, one that clearly differentiates it from screening. A central aspect of this distinction is the disassociation of case-finding from one of the taken-for-granted *whats* of screening: its population-based character. Case-finding is instead connected to another *what*: the application of a test on a case-by-case basis, according to a particular clinician’s clinical judgment.

Having made this distinction, Ranson et al. surprisingly go on to reconnect case-finding with screening by arguing that “the criteria for assessing evidence for screening proposals […] also apply to case-finding” [28]. Although case-finding is clearly distinct from screening, it should be assessed using the same standards as the latter, they argue. Thus, while disassociating case-finding from the *what* of population-based screening, Ranson et al. reconnect it with the *how* specified by the screening criteria. In their assessment of case-finding for dementia against the (UK NSC) screening criteria, they conclude that it does not fulfil the criteria and should currently not be offered [28].^6^

Yet another distinction is also sometimes made: between “passive” and “active” case-finding [see e.g., 30, 31]. Mate et al. define “passive” case-finding as an activity in which “patients are evaluated for dementia because they or a caregiver bring a memory/cognition concern to their GP, or because their GP raises the issue based on their clinical judgement” [20]. “Active” case-finding, on the other hand, they state, “specifies an opportunistic dementia assessment of ‘at-risk’ patients based on a number of factors including vascular risk factors, Parkinson’s disease and learning disabilities, in addition to subjective memory complaint” [20]. The difference between active and passive case-finding, according to them, lies in the level at which the decision to test is made: if this is a clinical level the case-finding is passive, while if an “at-risk” population has been identified on the level of the health system the case-finding is active, and allows for the testing of individuals before they experience symptoms or show signs of disease. Mate et al.'s definition of passive case-finding is close to Ranson et al.’s definition, and results in a clear distinction from screening. This clear distinction (between the *whats*) leads Mate et al., unlike Ranson et al., to propose that passive case-finding should not be assessed against the same criteria as screening [20]. Their conclusion is that passive case-finding is already taking place in primary care, and that the benefits of introducing a screening program in its place would not outweigh the potential harm associated with the latter [20]. In other words, they argue that a difference in the *whats* should entail a difference in the *hows*.

Two contrasting effects of the definition of active case-finding proposed by Mate et al. can be identified. On the one hand, the definition makes it more difficult for them to uphold the distinction from screening—since active case-finding, in their use of the term, retains some of the population-based characteristics associated with screening. For this reason, Ranson et al. would likely conceptualize active case-finding as a form of screening, while the UK Department of Health can argue that this is the form their case-finding scheme takes. On the other hand, the definition helps set active case-finding apart from screening. Rather than a test that is systematically offered to everyone in the predefined group, active case-finding is offered when the opportunity arises. This opportunistic character is a defining feature also of passive case-finding. Above, we saw how Ranson et al. defined case-finding as an “opportunistic investigation” [28].

Here another conceptual complication arises, namely, how to distinguish case-finding from “opportunistic screening”. The term “opportunistic screening” is an example of a concept that is used to *distinguish between different types of screening.* When it is used, the population-based character that is ordinarily implicit in definitions of “screening” comes to the forefront. As Speechley et al. have shown, however, the concept of opportunistic screening is often used synonymously with case-finding in the scientific literature [33].^7^ Why this is so becomes evident when we look more closely at some of the definitions of opportunistic screening that are offered. Opportunistic screening is generally defined in two slightly different ways. In some contexts, the emphasis is put on the actor asking for or offering the test, as in the definition used by the New Zealand National Screening Unit, which states that opportunistic screening “happens when someone asks their doctor or health professional for a check or test, or a check or test is offered by a doctor or health professional” [23]. In other contexts, the emphasis is put instead on the setting in which the test is administered, as in the definition used by Ashford et al., in which opportunistic screening is understood as an activity applied “to individuals who for other reasons [than screening] come to a setting where screening might occur” [2]. Although less detailed, these definitions have much in common with the conceptualizations of passive and active case-finding discussed above. The definition of opportunistic screening used by the New Zealand National Screening Unit overlaps with the definition of passive case-finding, since the decision to administer the test is taken by the actors involved in a clinical encounter. The definition of opportunistic screening proposed by Ashford et al. overlaps with the definition of active case-finding, since the testing is a result of a pre-existing structure of a particular healthcare institution, or of the healthcare system in general.

We see that it is difficult to separate the *whats* and the associated *hows* of case-finding and opportunistic screening. The choice between them and other concepts is ethico-politically charged. While “screening” might not be a proper term for the “case-finding” scheme that is used in the UK, since the testing is not offered universally to all citizens above the age of 75, evading comprehensive assessments by choosing the term “case-finding” instead of a concept that contains the term “screening” (such as “opportunistic screening”) is also questionable. We discuss below the ethico-political implications of such conceptualizations, and end the article by proposing a set of recommendations.

## Implications of the Conceptual Debate

Wilson and Jungner provided a wide definition of the concept of screening (*what*) in their influential WHO report, and they offered a detailed *how* in the form of their ten criteria. This *how* became coupled with a certain *what*, i.e. population-based screening, which led to a situation of conceptual experimentation. This is not surprising, if we consider the performative nature of concepts and practices.

First, if existing concepts are defined differently or entirely new concepts are formulated, novel possibilities may emerge. In our analysis, we saw how the concept of screening was redefined, divided into two, and distinguished from case-finding, all of which opened up particular possibilities for action. In the UK, such a conceptual approach made it possible to adopt a screening-like practice for dementia that would otherwise have been ethico-politically difficult, if not impossible, to realize. Here, the performativity of concepts, i.e. their ability to “make up” new “spheres of possibility”, as Hacking puts it [13], becomes strikingly visible.

Second, the reverse may also be the case; practices may influence concepts. An example of this was the argument put forward by Mate et al. that the concept of “passive case-finding” was needed to denote an already existing practice for which this concept would be fitting. In their view, passive case-finding is something other than screening, and should therefore be assessed on other grounds. This is an example of the “looping effect” that Hacking writes about, which is a consequence of the mutual dependence and influences that characterise the relationship between concepts and practices.

Thus, conceptualizations are inherently non-neutral. They are designed for specific purposes. What we aim for in this article, then, is not neutrality, but conceptual clarity. We want to contribute to greater openness and reflection about the motivations behind, and potential implications of, conceptualizations. A lack of clarity about the meaning of a concept and/or the absence of ethico-political considerations about its implications should not let it pass under the radar.

We wish to contribute to such clarity and reflection by providing a set of recommendations. Our ambition in the following section is not to solve the ethical dementia-screening/case-finding dilemma, but to present recommendations for the definitions of screening and related concepts, on the basis of the complexity that the case of screening for dementia exemplifies.

## Recommendations

We recommend that ethico-political criteria and systematic assessment procedures be devised for every form of “screening” practice, be it population-based screening, opportunistic screening, or active or passive case-finding. It should not be possible to evade ethical scrutiny by renaming a practice. This also means that Wilson and Jungner’s assessment criteria and the additional criteria identified by Andermann et al. are not directly transferable to population-based opportunistic screening or case-finding, or any other form of screening or case-finding.

We suggest that the term “population-based systematic screening” should be used for practices that target a predefined population and offer testing to it, without healthcare professionals making clinical judgements as to whether a specific individual should be offered the testing. In contrast, the term “case-finding” should be reserved for practices in which patients are selected on the basis of clinical judgment,^8^ on a case-by-case basis. Such case-finding may be more or less politically motivated, and may coincide with the common scenario in healthcare in which a patient seeks healthcare based on experienced symptoms. A distinguishing feature of case-finding is that clinical judgment is decisive for the decision about who should and who should not be offered the test. This understanding of case-finding excludes both the UK case-finding scheme and the USA Annual Wellness Visit, since they target a predefined population.

The distinction between population-based systematic screening and case-finding is founded on our conviction that there is a crucial difference between a test offered universally to a predefined population and a test offered by a particular clinician to a particular patient, based on the former’s judgment of whether the person in front of her would benefit from the test. This is so, even though the test instrument used may be the same in both situations. We hold that a clear distinction between screening and case-finding is needed, and, unlike Ranson et al., we argue that *different* ethico-political criteria must be applied in these two cases.

The screening criteria of Wilson and Jungner and of Andermann et al. are often used to ensure the viability of population-based screening practices, in which the discretion of individual clinicians should not affect the selection of patients. Were these criteria to be applied to case-finding, it would probably be difficult, if not impossible, to use the clinical judgment that is so central to the practice. It would, for example, be difficult for a clinician to argue that a particular patient would benefit from a test in cases in which the effectiveness of available treatments for the disease is debated. Thus, criteria for case-finding must be devised that ensure, rather than hamper, clinical judgment that is sensitive to the particularities of the unique patient. This does not mean that there cannot also be criteria that oblige clinicians to consider, for example, the absence or presence of at-risk populations, or general evidence relating to the potential benefits and harms of case-finding, in their assessment of whether a particular person should be offered a test.

Returning now to the concept of screening, we recommend that a distinction be made between what we have termed “population-based systematic screening” and “population-based opportunistic screening”. As seen in the previous paragraph, we recommend that the term “screening” is used exclusively for population-based practices (while we acknowledge that populations differ in size). However, whether the targeted population is offered the test systematically or opportunistically may still differ.

In population-based systematic screening, everyone in the predefined population is offered testing. Most often, this takes place by a letter in the mail. This form of screening is to be differentiated from population-based opportunistic screening, in which the members of the predefined population who “come to a setting where screening might occur” [2] are offered the test, when they seek healthcare for other reasons.

This is a crucial difference. The orderly and predictable nature of population-based systematic screening facilitates equal access to the test, consistent informed consent procedures, standardized evaluation protocols, and so on. In the less orderly and less predictable population-based opportunistic screening practice, these conditions are more difficult to fulfil. Population-based opportunistic screening must take into account the specific vulnerability of the patient who is treated for non-screening-related illness, and who might not be prepared to take a test. Therefore, we assert, this vulnerability should be reflected in a difference between the *how* and *what* of this type of screening. It should be possible, for example, to refrain from offering the test to certain patients.

We recommend that the term “population-based opportunistic screening” should be used for screening that is opportunistically offered and targets a predefined population. However, in this type of screening, while everyone within the target population is targeted by default, just as in population-based systematic screening, clinical judgment may here be used to decide who should *not* be offered the test. This conceptualization of opportunistic screening is different from that of the New Zeeland National Screening Unit. The latter is “case-finding”, according to our conceptualization.

One result of adopting these recommendations is that the term “active case-finding” would become redundant. What Mate et al. defined as “active case-finding” should instead be understood as population-based opportunistic screening, since it targets a predefined population. Further, the UK “case-finding scheme” is, to us, population-based opportunistic screening, due to its targeted population and opportunistic character. The USA AWV, in contrast, is offered universally to a target population, and therefore falls under the category of “population-based systematic screening”.

**Fig. 1:**
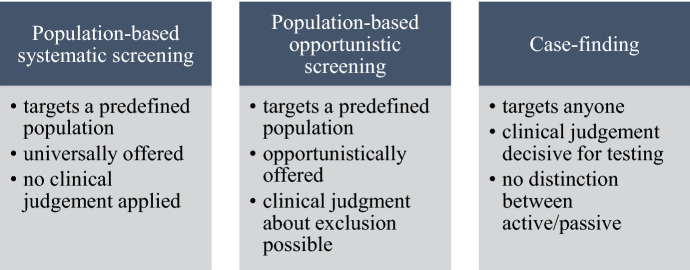
Summary of recommendations

Figure 1 summarises our recommendations.

## Conclusion

This paper had two aims. The first aim was to present an analysis of different conceptualizations of screening for dementia, and related terms, with a focus on how distinctions and delimitations make different courses of action possible. We showed how the debate about screening for dementia is thoroughly conceptual in character and that there is an intimate bond between the conceptual *what* and the practical *how* of screening. Ashford et al. uses a *redefinition* of screening to argue for the viability of population-based screening for dementia, while the use of the terms “case-finding” (in the UK) and “detection of cognitive impairment” (in the US) are examples of conceptual *distancing from the concept of screening* used to realize practices that would otherwise probably not have been possible. The last step of our analysis showed how “opportunistic screening” is used to make a *distinction* within the concept of screening, a distinction that increases rather than reduces the conceptual lack of clarity that characterises screening and related concepts.

The second aim of the article was to help reduce the lack of clarity that characterises screening and related concepts, by offering a set of recommendations. We recommend that a distinction be made between population-based systematic screening, population-based opportunistic screening, and case-finding. In our view, the use of these three terms allows for a clearer connection between the *what* of the concepts and the *how* of the practices, which may help to counteract what McCartney saw as a problem: the tendency to use conceptual experimentation in order to evade ethico-political scrutiny.

These recommendations do not exhaust all possible conceptualizations and practical materializations of screening, but they tackle some of its most central manifestations. The example of screening for dementia is illustrative, not primarily because it shares medical, practical, or technical features with other forms of screening, but because the debate about it to such a large extent takes the form of and results in a conceptual discussion and experimentation. It is when such conceptual discussion and experimentation take place that our results and recommendations are particularly applicable. One example could be the discussion in Sweden about screening for prostate cancer using PSA tests. In 2018, the Swedish National Board of Health and Welfare (Socialstyrelsen) recommended against population-based systematic screening for prostate cancer, on the ground that the benefits do not outweigh the harm [32]. To avoid so-called “Unorganized PSA testing” that might follow from this recommendation against screening, however, the National Board of Health and Welfare together with the Regional Cancer Center (RCC) issued recommendations on so-called “Organized Prostate Cancer Testing” (OPT), using PSA tests [29]. The aims of the recommendations were to standardize and streamline PSA testing, and to increase the knowledge about complementary diagnostic tests. In June 2018, the southernmost region in Sweden, Skåne, was the first region to introduce OPT, which was launched as a pilot project in the fall of 2020. Eventually, all men between the age of 50 and 74 will be offered testing. In our terminology, this practice constitutes screening, since it is population-based. Yet, the term screening is not used. Instead, it is supplemented by the words “organized” and “testing”. In this example, then, the practice is conceptually delineated from “screening”, on the one hand, and “unorganized testing”, on the other. This makes the debate about screening for prostate cancer and the recommendations about OPT illustrative examples of the intimate interplay between concepts and practices, for which our analysis is applicable. As we argue, if one is to understand and evaluate the ethico-political aspects and consequences of screening-like practices, this interplay must be examined.

Formulating comprehensive assessment criteria for each one of the forms of screening/case-finding discussed here is, however, beyond the scope of this article. Such criteria should not only evaluate the specificity and sensitivity of available test instruments, but also consider the implications of the practices for all persons who are tested, for healthcare professionals and the institutions in which they work, and for the society in which they take place. These aspects require a combination of qualitative and quantitative research and should be regularly evaluated once the screening/case-finding program has been introduced.

## Footnotes

1 Here we do not use the word “experimentation” in the natural scientific sense. Rather, we use it to denote the ways in which screening concepts are invented, redefined, and distinguished from each other.

2 These are not the only distinctions and delimitations that are made. In an article that reviewed existing conceptualizations of screening and screening-like practices, Speechley et al. found concepts such as “mass screening”, “multiphasic screening”, “periodical health examination”, “prescriptive screening” and “targeted screening” [33].

3 In the debate about screening and diagnosing dementia, the ideal of early detection has received some criticism. Mate et al., for example, argue that “a dementia diagnosis should be provided in a ‘timely’ fashion, that is appropriate for the circumstances of individual patients, rather than any particular disease stage” [20]**.** The need for a “timely” rather than early diagnosis is also emphasised in documents such as *Dementia Revealed* [5]**.** On the whole, however, these are exceptions. In national dementia strategies such as the Swedish [32], German, and French, as well as in a majority of scientific articles [e.g. 2,4,17,36], “early” detection and diagnosis is what is favoured.

4 While the UK National Screening Committee has discussed and recommended against population-based screening for dementia, other screening committees in other countries such as Sweden have not assessed population-based screening for dementia, and such screening is not part of the national dementia plan [outlined by 31].

5 In the absence of any detailed instructions on how to perform the cognitive assessment required during the AWV, several professional organizations have developed recommendations that build further on the ones provided by the CMS [19]. The Alzheimer’s Association, for example, has developed an algorithm that recommends a “brief structured cognitive assessment”, which includes the use of a cognitive test instrument (GPGCOG or Mini-Cog or MIS), if “signs/symptoms of cognitive impairment” are found during the observation and interview or if no knowledgeable informant is present [12].

6 This is indeed surprising, and we can only speculate about why they make this move. What this example clearly illustrates, however, is how the dynamic relationship between concepts and practices can open up a space of action for combining and recombining them in different ways.

7 See [6] for an example of such a use in the context of dementia.

8 Clinical judgment is a complex phenomenon, which may involve a medical professional’s actions and deliberation with regard to a particular patient during an extended period of time. As such, it may also involve shared decision-making with the patient or with other relevant persons. In this article, we use clinical judgment in a more narrow sense, denoting the professional’s situational assessment about whether they should offer a test to a particular patient.
